# The association between social capital and HIV treatment outcomes in South Africa

**DOI:** 10.1371/journal.pone.0184140

**Published:** 2017-11-09

**Authors:** Grace Musanse Mukoswa, Salome Charalambous, Gill Nelson

**Affiliations:** 1 School of Public Health, Faculty of Health Sciences, University of the Witwatersrand, Johannesburg, South Africa; 2 The Aurum Institute, Johannesburg, South Africa; National and Kapodistrian University of Athens, GREECE

## Abstract

**Background:**

HIV treatment has reduced morbidity and mortality. By 2012, it was estimated that 60.4% of eligible South Africans accessed antiretroviral treatment; however, treatment adherence and retention remain the greatest challenges. There is a growing belief that social capital, seen as "the features of social organization that facilitate cooperation for mutual benefit", is important in promoting HIV treatment retention. The aim of this study was to establish whether social capital is associated with HIV treatment outcomes.

**Methods and findings:**

This was a cross-sectional analysis of data from a cohort study that investigated how patient outcomes were linked to clinical characteristics, and included exploratory factor and logistic regression analysis. Data from 943 patients were analyzed. Outcomes for the analysis were visit non-adherence, unsuppressed viral load, and treatment failure. Sixteen percent of patients (n = 118) had unsuppressed viral loads; 19% (n = 179) were non-adherent; and 32% (n = 302) experienced treatment failure. Social capital had two dimensions that were described by two factors. There was no association between either factor and visit non-adherence. Social capital factor 1 was marginally associated with lower risks of unsuppressed viral load and treatment failure at 12 months (OR = 0.78; 95% CI = 0.58–1.03 and OR = 0.76; 95% CI = 0.62–0.93, respectively); but not with visit non-adherence (OR = 0.93; 95% CI = 0.71–1.22). After controlling for confounders, the odds of both unsuppressed viral load and treatment failure decreased with an increase in social capital factor 1.

**Conclusion:**

This study suggests that social capital, in terms of the number of groups to which an HIV-infected person belongs, the diversity of the groups, availability of child support, and time available for community projects, is protective against poor HIV treatment outcomes. Implementers and policy makers in the areas of HIV treatment and prevention need to consider the inclusion of social capital in the design of HIV/AIDS treatment program.

## Introduction

Social capital is important in promoting health globally. Putnam et al. [1] defined social capital as the “features of social organization, such as trust, norms and networks, which can improve the efficiency of society by facilitating coordinated action”. However, the World Bank (2001) defines it as networks of people deriving benefit from common interaction with each other [2]. The concept of social capital has been associated with health outcomes of people living with HIV/AIDS, a group of people considered globally to be a marginalized population [3].

HIV treatment program have measurably reduced morbidity and mortality [4]. South Africa has made remarkable progress in providing access to antiretroviral treatment (ART) and the country is currently implementing the largest ART program in the world [5]. The South African ARV program reached 34.7% of females and 25.7% of males at the time of the survey in 2011 [6], but treatment retention remains a key challenge [7].

Social relationships are influenced by external events. In the case of HIV, the greatest challenge may be that of stigma [8]. Social capital may reduce or increase both the perception and experience of stigma, thereby influencing HIV treatment adherence. The main pathways through which social networking might influence health outcomes include social support, social influence, social engagement, person-to-person contact, and access to material resources.

There is a growing belief that social capital is vital in promoting HIV treatment retention. Pronyk et al. [9] and others [10] have emphasized that high levels of social capital and community cohesion are necessary for successful HIV treatment implementation. Their beliefs were based on the ability to combat HIV-related stigma through interactions between individuals and their communities. They emphasized four themes based on interpersonal processes, the community structure environment, social disorder, and civic engagements.

The need to explore the association between social capital and HIV treatment outcomes has been suggested in several reports [10–14]. Wouters et al. [13] examined the relationship between bonding (such as having a treatment or emotional buddy) and bridging (access to a community health worker or social support) social capital, and the impact of different forms of social capital on patients' public disclosure. The study showed that bonding social capital was more effective in promoting public disclosure of HIV. Webel et al. [12] looked at the association between social capital and health status amongst people living with HIV/AIDS and found strong evidence to suggest that social capital can be used in influencing different health-related issues.

We aimed to establish whether social capital is associated with HIV treatment outcomes, particularly virological failure, in HIV patients on ART at 12 months in South Africa (see Box 1 for explanation of terms).

**BOX 1. Definitions of terms used in this study**

**Antiretroviral Treatment (ART):** A therapy used in treating HIV infection, usually given as a combination of three drugs—first line usually made up of two nucleoside reverse transcriptase inhibitors and one none-nucleoside reverse transcriptase inhibitor.

**Social Capital:** The features of social organisation, such as trust, norms and networks that can improve the efficiency of society by facilitating coordinated action.

**Visit non-adherence:** Fewer than 5 visits over the first 12 months after start of ART.

**Unsuppressed viral load:** >400 copies/mL at 12 months after start of ART.

**Treatment failure:** ≤ 400 copies/mL or missing viral load at 12 months after start of ART.

## Materials and methods

This was a cross-sectional analysis of data from a large cohort study [15] examining site-level determinants of HIV outcomes, which was conducted at private and non-governmental organization (NGO) clinics from January 2006 to December 2010. The study measured social capital in individuals and used cohort data on retention and virological suppression over 12 months in those individuals to determine the associations between outcomes and social capital.

### Participants

Clinics located in five provinces in South Africa (Gauteng, North West, Free State, Limpopo and Mpumalanga) were chosen if they had more than 80 patients that had initiated ART during the 12-month study period. Of the 34 clinics, 17 were located in towns, 14 in cities and three in rural areas. Private solo practitioners ran 25 clinics, six were private group practices, and three were NGO clinics.

The study participants consisted of HIV positive adults who were on ART for more than one month at the selected clinics from January 2006 to December 2010. Patients who attended a clinic visit (and were on ART for approximately 2 weeks to 2 months) during the study period for each of the selected clinics were included in the study (N = 1324).

### Procedures

Patients were interviewed as they exited from their first clinic visit, after giving written informed consent to participate in the primary study.

It is important to emphasize that no validated tool was available for measuring social capital at the time of the study. The questionnaire was drawn up from previous studies on ART adherence and perceptions around HIV and health [16], and on barriers and perceptions to access health services [17] in South Africa. Although the final questions regarding social capital were not validated, they were drawn from Statistics South Africa [18] and World Bank tools [19]. The two persons who designed the questionnaire had done extensive reading on social capital, and have since published on the topic using this tool [20,21].

Information was collected to determine social capital as defined by groups and networks; trust and solidarity; and collective action and cooperation. The questionnaires included questions concerning each of the three categories of social capital and scores were assigned to responses. For example, “groups and networks” included questions on the household member's participation in various types of social organizations, and relationships with close friends. For “trust and solidarity”, questions considered if, in general, people can be trusted, and the levels of trust toward neighbors, strangers and other service providers. For “collective action and cooperation”, questions were asked about how members of a community or household worked on joint projects and/or in response to a crisis.

Social capital was measured using eight categories (see Table 1), viz.

1. *number of groups*: the number of groups or organisations to which the participant belonged (e.g. church group, sports club, etc.). One point was allocated per group, with a maximum score of five.
2. *group diversity*: the diversity of the members of the different groups or organisations to which the participant belonged. One point was allocated per group, with a maximum score of six.
3. *friends*: the number of close friends that the participant had. One point was allocated per friend, with a maximum score of five.
4. *trust*: if the participant had trust in people in general. A score of zero was allocated for a negative response; five points was allocated for a positive response.
5. *level of trust*: the level of trust someone had towards a category of people or groups like neighbors, strangers, national leaders or police. Points were allocated from one to five, based on a Lickert scale (from ‘no trust’ to ‘complete trust’)
6. *time*: how much time someone had available to contribute to a community project. A score of zero was allocated to a negative response; five points was allocated to a positive response.
7. *availability of child support*: if someone was at hand to care for the participant’s child/children. Two points were allocated for a relative being available; five points were allocated for a person who was not a relative
8. *ability to borrow money* from someone, including friends and relatives. Points were allocated from one to five, based on a Lickert scale (from ‘definitely not’ to ‘definitely’).

**Table 1:** Categories of social capital measured on the questionnaire.

category no.	Category	Scoring	Max score
1	No. of groups	1 point per group	5
2	Group diversity	1 point per diverse group	6
3	Friends	1 point per friend	5
4	Trust	No = 0 Yes = 5	5
5	Level of trust	1 point per category of people or group	5
6	Time	No = 0 Yes = 5	5
7	Availability of child support	None = 0 Relative = 2 Other = 5	5
8	Ability to borrow money	Lickert scale (‘definitely not’ to ‘definitely’; 1–5	5

Because the Cronbach’s alpha for the eight categories was very low, viz. 0.23, indicating low internal consistency, exploratory factor analysis was carried out in order to find fewer variables to represent social capital.

Routine data that were collected as part of the program, from patients at each clinic visit after initiation of ART, included demographic information and laboratory electronics records (for CD4 and viral load). Baseline CD4 count was any CD4 test done within one month prior to start of ART. Changes in treatment were also included in the analysis.

The outcomes for the analysis were visit non-adherence, unsuppressed viral load, and treatment failure. Visit non-adherence was defined as fewer than five visits in the first year after starting ART. Visits following ART initiation were scheduled at 2 weeks, 6 weeks, 3 months, 6 months, 9 months and 12 months after the start of ART (seven visits in the first year, including the ART initiation visit). Unsuppressed viral load was defined as HIV RNA viral load >400 copies/mL at 12 months after the start of ART. HIV RNA was assayed using polymerase chain reaction (Amplicor HIV-1 Monitor Test, Roche Diagnostics, Basel, Switzerland). Treatment failure was defined as either no viral load result or viral load >400 copies/mL after 12 months. We also ran a sensitivity analysis with different cut off values for treatment failure, i.e. <1000 copies/mL and <200 copies/mL. The three outcomes were dichotomized into visit adherence or non-adherence; suppressed or unsuppressed viral load; and treatment failure yes or no, respectively.

Social capital was the main predictor variable. Potential confounders were age, gender, education level, employment status, socio-economic status (including composition of residence), wealth/income, and health (having medical insurance).

### Statistical analysis

Data were analyzed using Stata version 12. Odds ratios in a logistic regression model were calculated for categorical variables and, where a trend was evident, a chi-square test for trend was conducted. The main variables of interest were social capital, defined by the two factors, and the three treatment outcomes. Variables that caused the effect sizes of the social capital factors to change by 10% or more were included as confounders of the three treatment outcomes in the final model. Associations between social capital and the treatment outcomes were also analyzed.

### Ethical approval

This study was approved by the University of the Witwatersrand Research Ethics Committee (certificate number: M130249).

## Results

**Fig 1:**
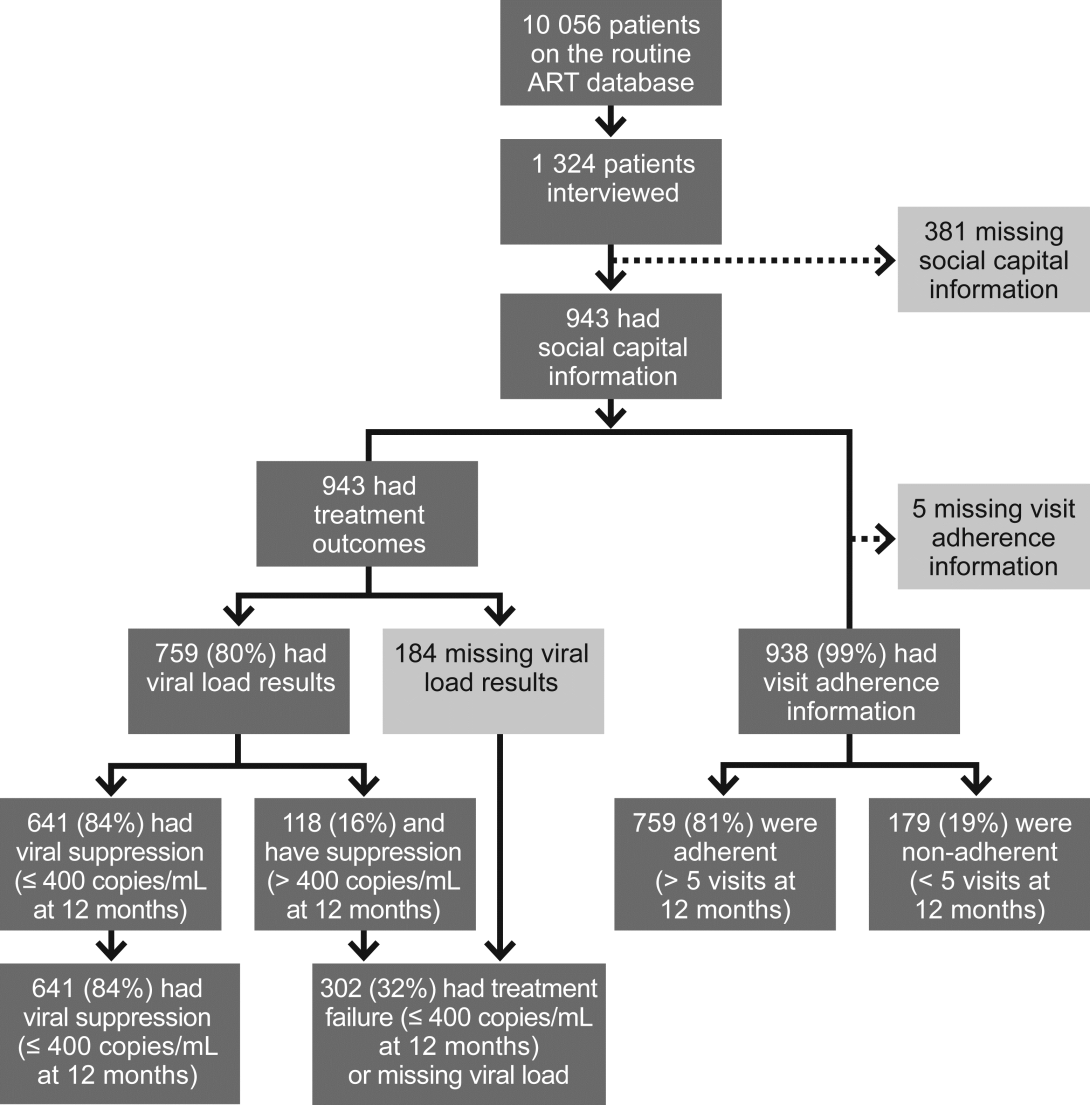
Number and proportions of participants in the study at various stages.

Fig 1 shows the numbers and proportions of participants in the study at various stages. There were 10 056 patients on the program with records available on the routine ART database. Of the 1 324 total HIV-positive patients interviewed, 943 (71.2%) had social capital information recorded. As shown in Table 2, females comprised 70.6% of the study population (n = 665). The average age of participants was 40 years (SD±8.9). The majority were single (n = 418; 44.4%), and most were unemployed (n = 533; 56.6%) and of medium socio-economic status (n = 257; 27.3%).

**Table 2:** Demographic characteristics of study participants (N = 943).

Variable	n	%
Gender		
Male	277	29.4
Female	665	70.6
Missing	1	0
Age(years)		
<30	100	10.6
30–40	414	44.0
>40	428	45.4
Missing	1	0
Marital status		
Married	242	25.7
Never married/Single	418	44.4
Widow/widower	109	11.6
Living together/partner	99	10.5
Divorced	74	7.9
Missing	1	0
Occupation		
Unemployed	533	56.6
Employed	409	43.4
Missing	1	0
Education		
Primary	93	10.0
Secondary	463	49.2
Undergraduate	386	41.0
Missing	1	0
Socio-economic status		
High	131	31.9
Medium	257	62.5
Low	23	5.6
Missing	532	56.6

Of the 943 patients with social capital information, all had treatment outcome data available. Most had viral load results recorded at 12 months (n = 759; 80.1%) and data on visit adherence at 12 months (n = 938; 99.9%). Of the 759 with viral load results, 641 (84.5%) had suppressed viral loads; 759 of the 938 (80.9%) were adherent (≥5 visits in the first year), and 302 (32%) of the 943 had treatment failure.

Factor analysis suggested that social capital is explained by two factors (Table 3). Factor 1 was emphasized by higher loadings (> 0.3) of group diversity, the number of groups to which the study participant belonged, availability of child support, and time available for community projects. Factor 2 was emphasized by close friends, trust, level of trust, the ability to borrow money, and time available for community projects.

**Table 3:** Results of factor analysis, showing loadings.

Variable score	Loading factor 1	Loading factor 2
Group diversity	0.84	0.02
No. of groups	0.85	0.01
Friends	- 0.22	0.55
Trust	0.05	0.63
Ability to borrow money	-0.10	0.43
Availability of child support	0.31	0.24
Time	0.35	0.33
Level of trust	0.13	0.69

Mean social capital scores at baseline are reported in Table 4. Males scored lower on factor 1. Those younger than 30 scored lower on factor 1 and higher on factor 2. Those living together scored higher on factor 1. Those employed scored higher on factor 1. Those with primary and secondary education scored lower on both factors. Those of low socio-economic status scored higher on factor 2.

**Table 4:** Mean social capital scores at baseline.

Variable	N	Factor 1 Mean (sd)	Factor 2 Mean (sd)
Gender			
Male	111	-0.20 (1.06)	0.01 (1.08)
Female	336	0.06 (0.97)	-0.001 (0.95)
Age (years)			
<30	39	-0.47 (0.91)	0.23 (0.94)
30–40 >40	199 209	0.02 (1.01) 0.06 (0.99)	-0.04 (1.04) 0.003 (0.97)
Marital Status			
Married	117	0.06 (1.02)	0.04 (0.92)
Never married/ single	205	-0.15 (0.98)	-0.04 (1.004)
Widow/widower	55	0.13 (0.94)	0.02 (0.94)
Living together/ partner	37	0.31 (1.14)	0.17 (1.10)
Divorced	33	0.13 (0.89)	-0.04 (1.12)
Occupation			
Unemployed	263	-0.15 (0.92)	-0.01 (0.99)
Employed	184	0.21 (1.07)	0.02 (1.02)
Education			
Primary	49	-0.15 (1.04)	-0.16 (1.07)
Secondary	213	-0.05 (0.98)	-0.02 (0.94)
Tertiary	185	0.09 (1.01)	0.08 (1.05)
Socio-economic status			
High	131	-0.07 (1.05)	0.06 (1.02)
Medium	256	0.11 (0.98)	-0.02 (0.01)
Low	23	0.04 (0.83)	0.31 (0.83)

Tables 5 and 6 show the results of the analysis of the associations of demographic factors with the different HIV outcomes (visit non-adherence, unsuppressed viral load and treatment failure).

**Table 5:** Univariable logistic regression analysis for HIV treatment outcomes.

Variable	Visit non Adherence^1^		Treatment Failure^2^		Unsuppressed viral load^3^	
	OR	95% CI	OR	95% CI	OR	95% CI
Social capital Factor 1 Factor 2	0.93 0.99	0.71–1.22 0.76–1.30	0.76 0.96	0.62–0.93 0.79–1.17	0.78 1.03	0.58–1.03 0.79–1.36
Gender						
Male	1 (Ref)		1 (Ref)		1 (Ref)	
Female	0.96	0.67–1.37	0.94	0.70–1.27	1.05	0.68–1.63
Age (years)						
<30	1 (Ref)		1 (Ref)		1 (Ref)	
30–40 >40	0.93 0.94	0.54–1.62 0.54–1.62	0.72 0.57	0.46–1.13 0.36–0.89	0.73 0.49	0.40–1.35 0.26–0.91
Marital Status						
Married	1 (Ref)		1(Ref)		1.(Ref)	
Never married/Single	1.16	0.77–1.75	1.15	0.82–1.62	1.80	0.77–1.75
Widow/Widower	1.37	0.79–2.39	0.62	0.36–1.05	1.07	0.79–2.39
Living together/Partner	0.59	0.29–1.20	1.11	0.68–1.82	1.37	0.29–1.20
Divorced	1.27	0.67–2.41	1.00	0.57–1.77	1.02	0.67–2.41
Occupation						
Unemployed	1 (Ref)		1 (Re)		1 (Ref)	
Employed	1.09	0.78–1.50	0.90	0.68–1.25	1.09	0.78–1.50
Education						
Primary	1(Ref)		1 (Ref)		1 (Ref)	
Secondary	1.22	0.69–2.16	1.30	0.81–2.12	1.22	0.69–2.16
Tertiary	0.88	0.49–1.58	1.05	0.64–1.73	0.88	0.49–1.58
Socio-economic status						
Low	1(Ref)					
Medium	-	-	0.69	0.29–1.66	0.45	0.16–1.26
High	1.70	0.94–2.94	0.96	0.39–2.38	0.50	0.18–1.48
NRTI regimen** Zidovudine (AZT) Tenofovir Stavudine (D4T)	1 (Ref) 0.55 0.63	0.35–0.88 0.17–2.43	1 (Ref) 1.31 1.36	0.83–2.07 0.42–4.32	1 (Ref) 0.85 0.95	0.48–1.53 0.19–4.76
NNRTI regimen*** Efavirenz Nevirapine	1 (Ref) 0.96	0.69–1.34	1(Ref) 1.28	0.97–1.69	1 (Ref) 1.57	1.04–2.36

^1^Less than 5 visits at 12 months

^2^ > 400 copies/mL or missing viral load

^3^ > 400 copies/mL at 12 months

** Nucleosides reverse transcriptase inhibitor

*** Non-nucleosides reverse transcriptase inhibitor

OR = Odds ratio, CI = Confidence interval

**Table 6:** Multivariable logistic model showing association between social capital and the three treatment outcomes.

Covariate	Visit non-adherence		Treatment failure		Unsuppressed viral load	
	AOR	95% CI	AOR	95% CI	AOR	95% CI
Social capital						
Factor 1	0.87	0.66–1.15	0.73	0.60–0.90	0.70	0.52–0.94
Factor 2	0.96	0.73–1.27	0.96	0.78–1.18	0.90	0.68–1.21
Age						
<30	1 (ref)		1(ref)		1(ref)	
30–40	1.25	0.44–3.53	0.67	0.32–1.40	1.06	0.36–3.11
>40	1.11	0.39–3.17	0.71	0.35–1.48	1.07	0.36–3.16
NNRTI						
First line	1 (ref)		1 (ref)		1 (ref)	
Second line	1.69	0.96–2.99	1.28	0.85–1.94	1.55	0.86–2.77

AOR = Adjusted odds ratio

### Visit non-adherence

In the univariable analysis, the use of tenofovir in the regimen was associated with visit non-adherence (0R = 0.55; 95% CI = 0.35–0.88). There was no association between non-adherence and either of the social capital factors. In the multivariable model, none of the variables was associated with visit non-adherence.

### Treatment failure

Being older than 40 years appeared to be protective against treatment failure (OR = 0.57; 95% CI = 0.36–0.89). The statistical model showed that the odds of treatment failure decreased with increasing social capital factor 1, i.e. the greater the group diversity, the more groups to which one belonged, and the greater the availability of child support, the lower the risk of treatment failure. There was no evidence of an association between social capital factor 2 (the number of friends a person had, the level of trust, the ability to borrow money, and time available for community projects) and treatment failure.

The sensitivity analysis using cut off values for treatment failure of <1000 copies/mL and <200 copies/mL did not change the statistical significance of the association between treatment failure and either social capital factor.

### Unsuppressed viral load

Being older than 40 years was protective against unsuppressed viral load (OR = 0.49; 95% CI = 0.26–0.91 and OR = 0.57; 95% CI = 0.38–0.86, respectively). However, the following factors were associated with an increased risk of having an unsuppressed viral load: being single (OR = 1.80; 95% CI = 1.07–3.03), and use of a nevirapine in the regimen (OR = 1.57; 95% CI = 1.04–2.36). When the model was adjusted for age and whether or not the patient was using a second line of treatment, there was an association between factor 1social capital and unsuppressed viral load (AOR = 0.70; 95% CI = 0.52–0.94).

## Discussion

This is the first study to measure the association between social capital and ART outcomes. Social capital factor 1 was associated with a lower risk of unsuppressed viral load and a lower risk of treatment failure at 12 months, but not with visit non-adherence. After controlling for confounders, the odds of both unsuppressed viral load and treatment failure decreased with increased social capital. This study is the first to show that social capital is linked to a lower risk of having an unsuppressed viral load for individuals on ART. In addition, we found that demographic factors associated with high social capital were older age and being employed.

These findings are in keeping to those from studies by Cattell et al. [22] and Kawachi et al. [23] who postulated that people with increased social capital cope better with stress and live longer than those with low social capital. A study from the US by Sapp et al. [24] provided evidence that social capital has produced clear benefits and has significantly contributed to the well-being of people with chronic diseases in communities. They reported that people who were members of religious organizations or who benefited from employment also enjoyed the extended benefits provided by these factors through social engagements. A typical example is workplace social capital where various social support, such as information exchange, cash loans and advice are available, has been known to serve as a buffer of job stress. This is supported by a study conducted in Finland on the association between workplace social capital and the onset of depression. Findings suggested that low individual level social capital at work is associated with the onset of depression [25].

Individuals with middle and higher social capital had a lower risk of treatment failure. This situation is similar to that reported in a study on cancer patients in the US [20]. By talking about their conditions, patients were able to overcome their ailments. They derived encouragement from members in their networks and groups and advice on diet and physical exercise. They also developed self-esteem and confidence. This knowledge may guide program implementers in designing program.

Social capital is a difficult concept to understand as it is measured in different ways and has a variety of definitions. Several studies have identified useful proxy measurements for social capital using different types and combinations of qualitative, quantitative and comparative research methodologies. This is one of the few studies that have looked at social capital and HIV treatment outcomes in South Africa, and the only one that has used a method of scoring social capital to show its effect on HIV/AIDS patients with regard to treatment outcomes.

Our study had some limitations. The first was the timing of the interviews. Social capital was measured at one-time point, but outcomes were measured at 12 months after the start of ART. There was an assumption that the social capital measured at the starting point remained constant while the person was on ART. Nevertheless, the study design provided prospective data on a sample of patients that would not have been available in a cross-sectional study. Another limitation was that we included only patients who were at the study clinics and available for interview. We are therefore likely to have overestimated treatment success due to selection bias. However, our study was not aimed at understanding treatment success but rather at understanding social capital and its effect on treatment success. In addition, as we did not have validated tools for social capital, we used modified social capital scores. This made it difficult to compare the findings with those from other studies.

We used visit adherence as a proxy for adherence to treatment as early studies show significant impact of social responsibility upon treatment adherence. We also included viral suppression, as this is the gold standard used to measure treatment adherence. We used a composite outcome of unsuppressed viral load and missing viral load or no viral load result to account for those who did not return for the viral load results as treatment failure. We assumed that the reason for missing viral load results was that patients did not return for their viral load visits, but there might have been other reasons, such as problems with the laboratory and the data management system.

At the same time, our review of different studies where most of researcher points on several gaps concerning validity of social capital scores. We did not have validated tools for social capital. In this study, we assumed that the reason why higher social capital was associated with older age could be that our study included 18 years and older. Further research should be done in younger groups and using validated tools.

## Conclusion

The results of this study show that, as the level of social capital increases, treatment outcomes improve. Most probably, this is linked to having the practical means to assist with the ability to continue taking treatment, assistance with child support, being part of a group or being part of a community project. In addition, it may be that HIV-infected people with high social capital tend to develop confidence, and that support results in improved treatment adherence and retention in the programme.

Implementers and policy makers involved in HIV treatment and prevention need to consider social capital in the design of HIV/AIDS treatment programmes and encourage support groups and other mechanisms for improving social capital. The association between social capital and employment also provides further evidence for the benefits of increased employment opportunities.

## Supporting information

S1 File Social capital questions contained in questionnaire used in cohort study. (DOCX)

S2 File Dataset1 used in analysis. (DTA)

S3 File Dataset2 used in analysis. (DTA)

S4 File Dataset3 used in analysis. (DTA)
