# Supplementary material for: The association between social capital and HIV treatment outcomes in South Africa
Source: PLoS One. 2017 Nov 9;12(11):e0184140. doi: 10.1371/journal.pone.0184140 (PMC5679596; doi:10.1371/journal.pone.0184140)
Supplement: S1 File — (DOCX) [file pone.0184140.s001.docx]

**QUESTIONS PERTAINING TO SOCIAL CAPITAL**

Questions relating to Social Capital, incorporated in questionnaire administered by AURUM for study to Determine Site-level Factors Which May Determine Clinical Outcomes on Antiretroviral Therapy (ART) in Patients Attending Primary Health Clinics in South Africa.

“I would like to start by asking you about the groups or organizations, networks, associations to which you belong. These could be formally organized groups or just groups of people who get together *regularly* to do an activity or talk about things. Groups can be lik*e a religious group mosque / church / shul / temple, savings club, burial society, political party, trade union, sport club, youth group, volunteer with police/fire brigade; residence association, women’s group, school committee, traders group, music group”*

| Of how many such groups do you or members of household a member? ..................  Of all these groups to which you or members of your household belong, which one is the most important to you household?  _______________________________________________________ [Name of group / type] |
| --- |
| Thinking about the members of this group, are most of them of the same  RELIGION (1=Yes, 0=No)................................................................................................  GENDER (1=Yes, 0=No)..................................................................................................  RACE (1=Yes, 0=No)..........................................................................................................  ETHNIC/LINGUISTIC BACKGROUND (1=Yes, 0=No)........................................................  OCCUPATION (1=Yes, 0=No)..........................................................................................  EDUCATION BACKGROUND/QUALIFICATIONS) (1=Yes, 0=No)....................................... |
| About how many *close friends* do you have these days? These are people you feel at ease with, can talk to about private matters, or call on for help…………............................ |
| If you suddenly needed to borrow a small amount of money [RURAL: enough to pay for expenses for your household for one week; URBAN: equal to about one week’s wages], are there people beyond your immediate household and close relatives to whom you could turn to and would be willing and able to provide this money?..................................................  1= Definitely  2= Probably  3=Unsure  4=Probably not  5=Definitely not |
| If you suddenly had to go away for a day or two, who would take care of your children? (give answers that apply) …………………..……………………………….…  …….…………………………………………….…  1= Blood relative (brother, sister, father, mother)  2= Other relatives (e.g. in-laws)  3= A non-relative close friend  4= Neighbour  5= Work colleague  6= Member of a group you belong to  7= No one |
| Generally speaking, would you say that most people can be trusted (Yes=1, No=0?).................... |
| If a community project does not directly benefit you but has benefits for many others in the village/neighbourhood,  Would you contribute time to the project? (Yes=1, No=0?)...............................................  Would you contribute money to the project? (Yes=1, No=0?)...................................................... |
| What is the level of trust between in community and the following types of people or groups?  1=No trust  2=Low trust  3=Medium trust  4=High trust  5=Complete trust  Neighbours...........................................................................................................................  Local leaders........................................................................................................................  Strangers..............................................................................................................................  Newspaper/radio/TV............................................................................................................  Local government.................................................................................................................  Provincial government.........................................................................................................  National government............................................................................................................  National leaders....................................................................................................................  Police....................................................................................................................................  Security services ….............................................................................................................. |
